# Supplementary material for: A Revised Phylogenetic Classification for Viola (Violaceae)
Source: Plants (Basel). 2022 Aug 27;11(17):2224. doi: 10.3390/plants11172224 (PMC9460890; doi:10.3390/plants11172224)
Supplement: Supplementary file 1 [file plants-11-02224-s001.zip › supplementary/File S1.pdf]

Violets of the World

Thomas Marcussen, Harvey E. Ballard, Jiří Danihelka, Ana R. Flores, Marcela V. Nicola, John M. Watson

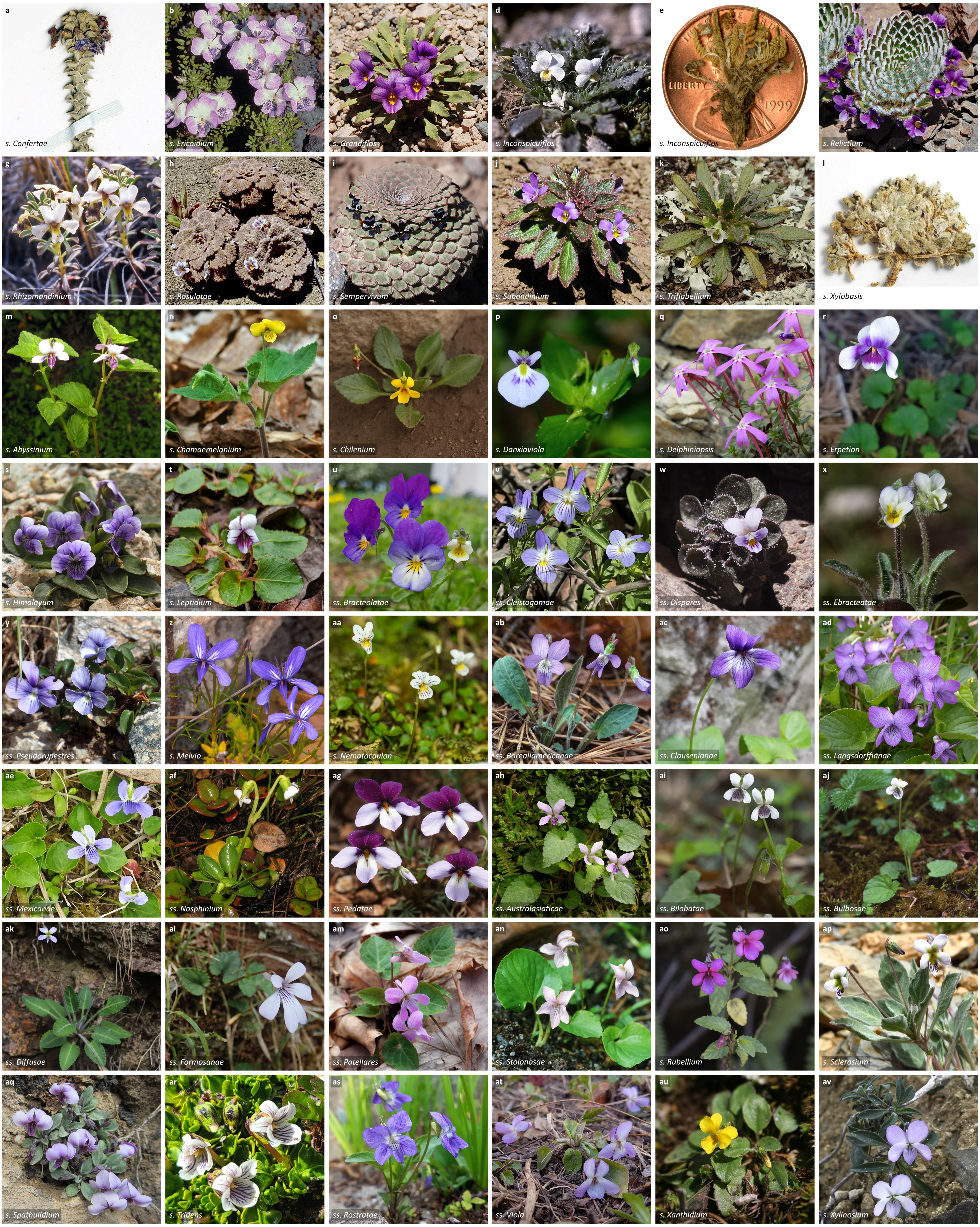

Photos of representative members of the sections and subsections of *Viola*. — **a–l**. Subg. *Neoandinium*. — **m–av**. Subg. *Viola*. — **a. Sect. *Confertae***: *V. nassauvioides*. — **b. Sect. *Ericoidium***: *V. fluehmannii* (photo © John M. Watson). — **c. Sect. *Grandiflos***: *V. cheeseana* (photo © John M. Watson). — **d. Sect. *Inconspicuiflos***: *V. weibelii* (photo © John M. Watson). — **e. Sect. *Inconspicuiflos***: *V. lilliputana* (photo © Harvey E. Ballard). — **f. Sect. *Relictium***: *V. dandoisiorum* (photo © John M. Watson). — **g. Sect. *Rhizomandinium***: *V. escondidaensis* (photo © John M. Watson). — **h. Sect. *Rosulatae***: *V. volcanica* (photo © Ana R. Flores). — **i. Sect. *Sempervivum***: *V. atropurpurea* (photo © John M. Watson). — **j. Sect. *Subandinium***: *V. subandina* (photo © Ana R. Flores). — **k. Sect. *Triflabellium***: *V. triflabellata* (photo © Ana R. Flores). — **l. Sect. *Xylobasis***: *V. beati* (photo © Ana R. Flores). — **m. Sect. *Abyssinium***: *V. abyssinica* (photo © Robert v. Blittersdorff). — **n. Sect. *Chamaemelanium***: *V. pubescens* (photo © Kim Blaxland). — **o. Sect. *Chilenium***: *V. maculata* (photo © Instituto de Botánica Darwinion). — **p. Sect. *Danxiaviola***: *V. hybanthoides* (photo © Qiang Fan). — **q. Sect. *Delphiniopsis***: *V. cazorlensis* (photo © Santiago Martín-Bravo). — **r. Sect. *Erpetion***: *V. banksii* (photo © Dewald du Plessis). — **s. Sect. *Himalayum***: *V. kunawurensis* (photo © Vladimir Epiktetov). — **t. Sect. *Leptidium***: *V. boliviana* (photo © Sam Wilson). — **u. Sect. *Melanium*, subsect. *Bracteolatae***: *V. tricolor* and *V. arvensis* (photo © Thomas Marcussen). — **v. Sect. *Melanium*, subsect. *Cleistogamae***: *V. rafinesquei* (photo © Mary Ann Yaich). — **w. Sect. *Melanium*, subsect. *Dispares***: *V. dyris* (photo © Alan Keohane). — **x. Sect. *Melanium*, subsect. *Ebracteatae***: *V. parvula* (photo © Albert Keshet). — **y. Sect. *Melanium*, subsect. *Pseudorupestres***: *V. nummulariifolia* (photo © Sylvain Piry). — **z. Sect. *Melvio***: *V. decumbens* (photo © Magriet Brink). — **aa. Sect. *Nematocaulon***: *V. filicaulis* (photo © Andrew Townsend). — **ab. Sect. *Nosphinium*, subsect. *Borealiamericanae***: *V. fimbriatula* (photo © Kim Blaxland). — **ac. Sect. *Nosphinium*, subsect. *Clausenianae***: *V. clauseniana* (photo © Thomas Marcussen). — **ad. Sect. *Nosphinium*, subsect. *Langsdorffianae***: *V. langsdorffii* (photo © Jonathan Goff). — **ae. Sect. *Nosphinium*, subsect. *Mexicanae***: *V. nannei* (photo © Neptalí Ramírez Marcial). — **af. Sect. *Nosphinium*, subsect. *Nosphinium***: *V. maviensis* (photo © Karl Magnacca). — **ag. Sect. *Nosphinium*, subsect. *Pedatae***: *V. pedata* (photo © Kim Blaxland). — **ah. Sect. *Plagiostigma*, subsect. *Australasiaticae***: *V. sumatrana* (photo © Mário Duchoň). — **ai. Sect. *Plagiostigma*, subsect. *Bilobatae***: *V. hamiltoniana* (photo © Toshihiro Nagata). — **aj. Sect. *Plagiostigma*, subsect. *Bulbosae***: *V. tuberifera* (photo © Masashi Igari). — **ak. Sect. *Plagiostigma*, subsect. *Diffusae***: *V. huizhouensis* (photo © Yanshuang Huang). — **al. Sect. *Plagiostigma*, subsect. *Formosanae***: *V. formosana* (photo © Kuan-Chieh (Chuck) Hung). — **am. Sect. *Plagiostigma*, subsect. *Patellares***: *V. tokubuchiana* (photo © Masashi Igari). — **an. Sect. *Plagiostigma*, subsect. *Stolonosae***: *V. pluviae* (photo © Kim Blaxland). — **ao. Sect. *Rubellium***: *V. rubella* (photo © Pablo Silva). — **ap. Sect. *Sclerosium***: *V. cinerea* (photo © Jerome Viard). — **aq. Sect. *Spathulidium***: *V. pachyrrhiza* (photo © Dieter Zschummel). — **ar. Sect. *Tridens***: *V. tridentata* (photo © larsonek). — **as. Sect. *Viola*, subsect. *Rostratae***: *V. canina* (photo © Thomas Marcussen). — **at. Sect. *Viola*, subsect. *Viola***: *V. collina* (photo © Thomas Marcussen). — **au. Sect. *Xanthidium***: *V. flavicans* (photo © Instituto de Botánica Darwinion). — **av. Sect. *Xylinosium***: *V. arborescens* (photo © Abdelmonaim Homrani Bakali). — All images used in this figure were cropped and gamma-corrected. — Links to the online sources for the images used in this figure under a creative commons (CC) licence: *V. abyssinica*, http://www.westafricanplants.senckenberg.de/root/index.php?page_id=47&id=2203#image=22051, © Robert v. Blittersdorff; *V. arborescens*, https://www.teline.fr/en/photos/violaceae/viola-arborescens#photo-7, © Abdelmonaim Homrani Bakali, CC BY-NC 4.0; *V. nummulariifolia*, https://www.inaturalist.org/observations/89240299, © Sylvain Piry, CC BY-NC 4.0; *V. banksii*, https://inaturalist.nz/observations/25048808, © Dewald du Plessis, CC BY-NC 4.0; *V. boliviana*, https://ecuador.inaturalist.org/photos/57308380, © Sam Wilson, CC BY-NC; *V. cazorlensis*, https://www.inaturalist.org/observations/109331467, © Santiago Martín-Bravo, CC BY-NC 4.0; *V. cinerea*, https://www.inaturalist.org/observations/65108391, © Jerome Viard, CC BY-NC 4.0; *V. decumbens*, https://www.inaturalist.org/observations/10807224, © Magriet Brink, CC BY-NC 4.0; *V. filicaulis*, https://www.inaturalist.org/observations/67950856, © Andrew Townsend, CC BY-NC; *V. formosana*, https://www.inaturalist.org/observations/25305342, © Kuan-Chieh (Chuck) Hung, CC BY-NC 4.0; *V. hamiltoniana*, https://www.inaturalist.org/observations/74528783, © Toshihiro Nagata, CC BY-NC 4.0; *V. langsdorffii*, https://www.inaturalist.org/observations/73470330, © Jonathan Goff, CC BY-NC-SA 4.0; *V. maviensis*, https://www.inaturalist.org/observations/39272209, © Karl Magnacca, CC BY-NC 4.0; *V. nannei*, https://www.inaturalist.org/observations/6527547, © Neptalí Ramírez Marcial, CC BY-NC; *V. rafinesquei*, https://www.inaturalist.org/observations/111043498, © Mary Ann Yaich, CC BY-NC 4.0; *V. rubella*, https://www.inaturalist.org/observations/93725094, © Pablo Silva, CC BY-NC 4.0; and *V. tridentata*, https://www.inaturalist.org/observations/103467450, © larsonek, CC BY-NC 4.0. — Links to the relevant CC licences: CC BY 4.0 (https://creativecommons.org/licenses/by/4.0/), CC-BY-NC and CC BY-NC 4.0 (https://creativecommons.org/licenses/by-nc/4.0/), CC BY-NC-SA 4.0 (https://creativecommons.org/licenses/by-nc-sa/4.0/).

Marcussen et al. 2022. A revised phylogenetic classification for *Viola* (Violaceae). *Plants* 11(17) 2224. https://www.mdpi.com/2223-7747/11/17/2224
